# Causal factors of cardiovascular disease in end-stage renal disease with maintenance hemodialysis: a longitudinal and Mendelian randomization study

**DOI:** 10.3389/fcvm.2024.1306159

**Published:** 2024-07-18

**Authors:** Dandan Tian, You Xu, Ying Wang, Xirui Zhu, Chun Huang, Min Liu, Panlong Li, Xiangyong Li

**Affiliations:** ^1^ Department of Hypertension, Henan Provincial People’s Hospital & Zhengzhou University People’s Hospital, Zhengzhou, China; ^2^ Department of Clinical Laboratory, The Third Affifiliated Hospital, Southern Medical University, Guangzhou, China; ^3^ Department of Medical Statistics, School of Public Health, Sun Yat-sen University, Guangzhou, China; ^4^ Department of Medical Imaging, Henan Provincial People’s Hospital & Zhengzhou University People’s Hospital, Zhengzhou, China; ^5^ The School of Electrical and Information Engineering, Zhengzhou University of Light Industry, Zhengzhou, China; ^6^ Department of Infectious Disease, the Third Affiliated Hospital of Sun Yat-sen University, Guangzhou, China

**Keywords:** end-stage renal disease, hemodialysis, cardiovascular disease, causal factors, diabetes

## Abstract

**Background:**

The risk factors of cardiovascular disease (CVD) in end-stage renal disease (ESRD) with hemodialysis remain not fully understood. In this study, we developed and validated a clinical-longitudinal model for predicting CVD in patients with hemodialysis, and employed Mendelian randomization to evaluate the causal 6study included 468 hemodialysis patients, and biochemical parameters were evaluated every three months. A generalized linear mixed (GLM) predictive model was applied to longitudinal clinical data. Calibration curves and area under the receiver operating characteristic curves (AUCs) were used to evaluate the performance of the model. Kaplan-Meier curves were applied to verify the effect of selected risk factors on the probability of CVD. Genome-wide association study (GWAS) data for CVD (*n* = 218,792,101,866 cases), end-stage renal disease (ESRD, *n* = 16,405, 326 cases), diabetes (*n* = 202,046, 9,889 cases), creatinine (*n* = 7,810), and uric acid (UA, *n* = 109,029) were obtained from the large-open GWAS project. The inverse-variance weighted MR was used as the main analysis to estimate the causal associations, and several sensitivity analyses were performed to assess pleiotropy and exclude variants with potential pleiotropic effects.

**Results:**

The AUCs of the GLM model was 0.93 (with accuracy rates of 93.9% and 93.1% for the training set and validation set, sensitivity of 0.95 and 0.94, specificity of 0.87 and 0.86). The final clinical-longitudinal model consisted of 5 risk factors, including age, diabetes, ipth, creatinine, and UA. Furthermore, the predicted CVD response also allowed for significant (*p *< 0.05) discrimination between the Kaplan-Meier curves of each age, diabetes, ipth, and creatinine subclassification. MR analysis indicated that diabetes had a causal role in risk of CVD (*β *=* *0.088, *p *< 0.0001) and ESRD (*β *=* *0.26, *p = *0.007). In turn, ESRD was found to have a causal role in risk of diabetes (*β *=* *0.027, *p *= 0.013). Additionally, creatinine exhibited a causal role in the risk of ESRD (*β *=* *4.42, *p *= 0.01).

**Conclusions:**

The results showed that old age, diabetes, and low level of ipth, creatinine, and UA were important risk factors for CVD in hemodialysis patients, and diabetes played an important bridging role in the link between ESRD and CVD.

## 1 Introduction

The end-stage renal disease (ESRD) is the leading cause of kidney-related mortality (1), accounting of over 1.2 million associated deaths annually worldwide (2). Patients with ESRD are candidates for kidney transplants (3), and have an overall 5-year survival rate reaching 80%−90% after transplants (4). However, maximizing access to transplants and reducing access disparities poses a challenge for patients receiving prompt kidney transplants (5). According to both the European and American ESRD management guidelines, hemodialysis (HD) is recommended as a method of renal replacement therapy (6). ESRD patients underlying HD enjoy a survival benefit (7), but nearly 20% of these patients would die from cardiovascular disease (CVD) within three years (8–10). For ESRD patients at high risk of CVD, timely clinical intervention holds the potential to prevent further deterioration of the viscera system and prolong survival time, particularly if targeted therapy can be initiated before ESRD progresses to the advanced stage (11, 12).

Several clinical factors are potential risk for ESRD who are likely to have CVD. Aging, hypertension, and diabetes can cause cardiovascular dysfunction in both the general population and HD patients (13–16). In addition, certain HD-specific risk factors, such as anemia, chronic inflammation, and metabolic disturbance, may be involved in the pathogenesis of CVD in HD patients (17). Conversely, adopting suitable lifestyle habits, such as a plant-based diet rich in fiber and low in protein, can reduce the CVD risk in HD patients (18, 19). Several predictive studies have been conducted to identify risk factors for CVD in HD patients and have shown promising results (20–23). However, these studies have only analyzed data from a single time point, which limits their ability to capture the developmental changes in risk factors over time and establish causal relationships among risk factors, CVD, and ESRD (24). Furthermore, the cross-sectional datasets used in these studies relied on simple regression techniques, focusing on population-averaged inference among variables. These reduce the performance of models and can lead to inconsistent results (25, 26). Thus, longitudinal data analysis is needed to identify clinical risk factors and improve the accuracy of CVD prediction in HD patients. This will ultimately contribute to more precise and effective clinical decision-making.

Mendelian randomization (MR) is a novel causality analysis method that utilizes the random allocation of genetic variants as instrumental variables to estimate the causal association between exposure and clinical outcomes (27, 28). MR provides robust causal inferences and is minimally affected by residual confounding. This is because genetic variants are randomly assigned from parents and normally fixed, making them unaffected by the outcomes and confounders. This random distribution of confounders is comparable to the randomization process in randomized controlled trials (29).

Therefore, in this research, longitudinal clinical data of HD patients would be analyzed to estimate and validate a prediction model. This model aims to serve as a useful tool for predicting CVD. Additionally, Mendelian randomization analysis was applied to evaluate the causal inferences between the risk factors and CVD in HD patients.

## 2 Materials and methods

### 2.1 The longitudinal population

In this retrospective study, a total of 609 HD patients were included from the hemodialysis centers of the Third Affiliated Hospital of Southern Medical University, Third Affiliated Hospital of Sun Yat-sen University, and Henan Provincial People's Hospital according between January 2015 to September 2022 with the following inclusion criteria: (1) regular hemodialysis for more than 12 months; (2) agegreater than 18 years old; (3) no prior history of CVD events before starting hemodialysis; (4) availability of clinical data matrix. Participants who met any of the following exclusion criteria were not included in this study: (1) pregnant; (2) currently suffering from ongoing infection; (3) diagnosed with malignancy. The study protocol was approved by the ethics committee in the Third Affiliated Hospital of Southern Medical University, the Third Affiliated Hospital of Sun Yat-sen University, and Henan Provincial People's Hospital.

### 2.2 The longitudinal data collection

At the start of the first hemodialysis therapy, demographic characteristics, medical history, and dialysis-related information, including dialysis frequency, dialysis volume, and vascular access was recorded by trained nurses. Before each hemodialysis session, patients underwent an assessment to establish the corresponding dialysis plan. This assessment primarily involved setting the blood flow rate, dialysis rate, and ultrafiltration rate based on factors such as body weight, heart rate, and blood pressure. During the hemodialysis process, real-time monitoring was conducted for the patient's blood pressure, heart rate, dialysate temperature, pre-pump arterial pressure, and post-pump arterial pressure. Additionally, nurses regularly monitored various indicators throughout the dialysis session and adjusted the dialysate flow rate according to the patient's specific condition. These measures ensure the safety and effectiveness of the hemodialysis procedure. Every approximately 4 months, overnight fasting blood samples were collected from each participant. Nineteen biochemical parameters, including blood routine (white blood cells (WBC), hemoglobin (HB), platelets (PLT), neutrophile granulocyte (NEU)), glucose, lipids (high density lipoprotein (HDL), low density lipoprotein (LDL), total cholesterol (CHOL), triglyceride (TG)), infectious disease antigen (HBsAg, hepatitis C virus (HCV), human immunodeficiency virus (HIV)), as well as liver and kidney function indicators (intact parathyroid hormone (ipth), neutrophile granulocyte (BUN), creatinine (CRE), uric acid (UA), calcium (Ca), phosphorus (P)), were measured at the laboratory and radiology of the three centers. Additionally, radiological examinations, including computed tomography, magnetic resonance angiography, and color ultrasound, were conducted annually for the diagnosis of CVD.

First non-fatal and fatal CVD events, including valvular heart disease, myocardial disease, arrhythmia extracorporeal defibrillation, cerebrovascular disease, and peripheral vascular disease after hemodialysis, were recorded (30, 31). CVD time was recorded from the first hemodialysis to the first CVD event. The data were collected from the three centers until September 30, 2022.

### 2.3 The longitudinal data analysis

The data was preprocessed using R software (version 4.2.2), including the following steps. Despite a standardized data collection form being applied, the presence of missing data in our data matrix was unavoidable due to measurements not being taken, failure to send or retrieve questionnaires, and errors in manual data entry. Firstly, the missing data rate was calculated for each subject at the identified time points. If a subject's missing data rate exceeded 30%, they were removed from the data matrix (32). Then, the missing values in the cleared data matrix were imputed using the “MissForest” R package. This package allows for the imputation of both continuous and categorical data, taking into account complex interactions and nonlinear relations (33).

To account for population-averaged inference and within-subject correlation between the repeated measures, we implemented a longitudinal model called generalized linear mixed model (GLMM) using IBM SPSS Statistics (version 26). The GLMM integrates both the fixed effects and random effects of predictors, which enhances the validity and reproducibility of the experimental findings (34). The fixed effects were used to measure whether candidate factors had an impact on the risk of CVD. However, considering that these factors were measured multiple times, there might be correlations introduced due to multiple measurements of the same observed variable. To account for this correlation and estimate the results more accurately, random effects was incorporated in our model to estimate whether the effect of risk factors varying at different time points. In the model, we used the logit link function to model the natural logarithm of the odds of CVD. The model could be expressed as the following form: log (*p*/1 - *p*) = X*β* + Z*γ* + ɛ, where *p* represented the *p* value of CVD; X was the design matrix for fixed effects; *β* represented the parameters vector for fixed effects; Z was the design matrix for random effects; *γ* represented the parameter vector for random effects; ɛ represented the vector of residual errors.

To compare the results with those of a traditional prediction model, we conducted a logistic regression model (LRM). The performance of both models was evaluated using a calibration curve and the area under the receiver operating characteristic curve (AUC). The calibration plot for the probability of objective response showed agreement between the predicted probabilities computed by the models and actual observations. The discrimination ability of the two models was measured by bootstrap-corrected AUCs.

The primary endpoint of interest was the probability of CVD, and the second endpoint of interest was the survival time. The former could be predicted by the GLMM. The variables which were significant (*p* < 0.05) in the GLMM were chosen as risk factors. To assess the survival time across different risk factors, the continuous variables such as age, ipth, CRE, and UA were binarization by the “survminer” package, an R package to determine the optimal cut point (35). The survival curves of each risk factor were constructed using the Kaplan-Meier method and compared with the log-rank test.

### 2.4 Mendelian randomization

The GWAS summary statistics of ERSD, CVD, diabetes, creatinine, and uric acid were searched and downloaded from the IEU Open GWAS project (https://gwas.mrcieu.ac.uk/), which includes large amounts of standard format of human GWAS summary statistics. There was no overlap between the participants in the GWAS study and the HD patients used for longitudinal analysis mentioned above. Some basic information of the population in the five GWAS statistics was shown in Supplementary Table S1.

Risk genetic variants [single-nucleotide polymorphisms (SNPs)] associated ($p\;<\;5\times 10^{-8}$ for CVD, diabetes, creatinine and uric acid and $p\;<\;5\times 10^{-6}$ for ESRD) with exposures were selected as instruments. The threshold for ESRD was relaxed because no SNP reached for $p\;<\;5\times 10^{-8}$ reference to the strategy applied in previous MR studies (36). SNPs were excluded in moderate linkage disequilibrium (*r*^2 ^> 0.001) to reduce bias due to genetic correlation. The linkage disequilibrium across these SNPs were calculated based on the European 1000-Genomers reference panel. SNPs who had association ($p\;<\;5\times 10^{-2}$) with outcomes were excluded to obtain the assumption of MR that instrumental variables are strongly associated with exposure and have no direct association with the outcome. We also excluded SNPs that were palindromic and had an intermediate allele frequency to further reduce potential bias and uncertainty, as previously described (37). Outliers were determined with respect to their contribution to global heterogeneity, quantified by Cochran's Q-statistic, using a significance threshold of 0.05 for *p*-value, 1 for the inverse variance weights, and 0.0001 for the tolerance threshold for performing the iterative inverse-variance weighted (IVW) approach (38).

The following bidirectional univariate MR analyses were conducted separately to determine the causal effects of each of genetically predicted risk factors (diabetes, creatinine, and uric acid) phenotype on CVD and ERSD risk, and then vice versa.

The IVW approach was applied as the main MR model, which provides most accurate estimate under the assumption that the instrumental variables were all valid (39). In order to account for potential heterogeneity and horizontal pleiotropy, three additional approaches for MR analyses including MR-Egger regression, weighted median and weighted mode methods, were applied. Cochran's Q test and MR-Egger intercept test were used to detect the heterogeneity and horizontal pleiotropy, respectively.

The odds ratios (ORs) and corresponding 95% confidence intervals (CSs) were used to assess the strength of causal associations. All *p* values were two-sided and this study used conventional significance level (*p *< 0.05). Genetic instrument selection and MR analyses were carried out using “TwoSampleMR” and “RadialMR” package in R.

## 3 Results

**Figure 1:**
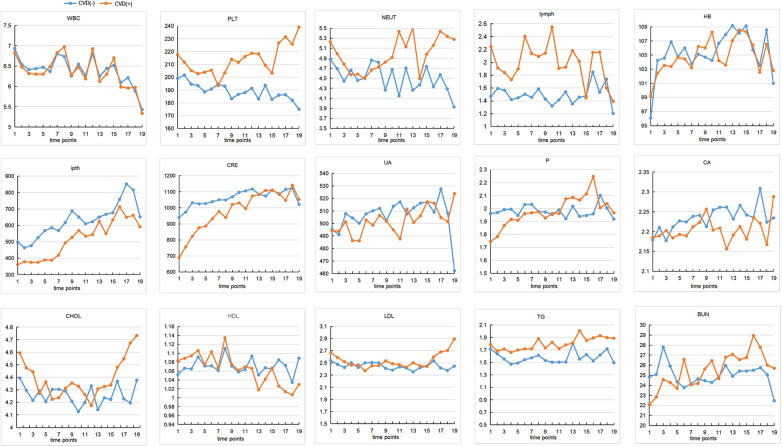
The changes of continuous biochemical variables. The interval between each time point on the horizontal coordinate is 4 months. The vertical axis represents the content of each variable. CVD, cardiovascular disease; WBC, white blood cells; HB, hemoglobin; PLT, platelets; NEU, neutrophile granulocyte; ipth, intact parathyroid hormone; BUN, blood urea nitrogen; CRE, creatinine; UA, uric acid; CA, calcium; P, phosphorus; CHOL, total cholesterol; TG, triglyceride; HDL, high density lipoprotein; LDL, low density lipoprotein.

Four hundred sixty-eight subjects were included after data cleaning, 221 from the Third Affiliated Hospital of Southern Medical University, 179 from the Third Affiliated Hospital of Sun Yat-sen University, and 68 from the Henan Provincial People's Hospital. Demographic information and biochemical parameters at baseline were shown in Table 1. 36.75% of the HD patients developed CVD within the following 4.7 years. Chi-square test showed that subjects with diabetes were more likely to have CVD (44.71% vs. 27.18%, *p* < 0.001). Two sample t-test showed that the CVD group had higher age (59.84 ± 14.13 vs. 52.07 ± 15.39, *p* < 0.001), WBC (7.67 ± 3.09 vs. 6.96 ± 2.99, *p* = 0.02), PLT (218.64 ± 72.74 vs. 198.86 ± 71.99, *p* = 0.005), lymph (2.12 ± 2.81 vs. 1.43 ± 1.16, *p* < 0.001), as well as lower ipth (361.67 ± 274.27 vs. 507.75 ± 430.19, *p* < 0.001), BUN (22.69 ± 11.16 vs. 25.07 ± 9.90, *p* = 0.02), CRE (709.31 ± 422.84 vs. 943.21 ± 340.93, *p* < 0.001), and *P* (1.76 ± 0.62 vs. 1.97 ± 0.60, *p* < 0.001), compared to the non-CVD group. No other group differences were found at baseline. The change over time of continuous biochemical parameters was shown in Figure 1.

**Table 1:** The demographic information and biochemical parameters at baseline.

			CVD(-)	CVD(+)	*χ*^2^/t	*p*
Sex	Male		193	116	0.58	0.45
	Female		105	54		
Age			52.07 ± 15.39	59.84 ± 14.13	5.41	<0.001
Vascular access (fistula/catheter)			246/52	127/43	3.65	0.056
amount			1,859.02 ± 731.79	2,075.49 ± 1,273.03	2.34	0.02
Primary renal disease		+	138	85	0.59	0.44
		-	160	85		
Hypertension		+	231	136	0.39	0.53
		-	67	34		
Diabetes		+	81	76	14.91	<0.001
		-	217	94		
Contagion		+	67	31	1.18	0.28
		-	231	139		
Combined disease		+	147	91	0.76	0.38
		-	151	79		
HBsAg		+	61	26	1.92	0.17
		-	237	144		
HCV		+	2	2	0.33	0.57
		-	296	168		
Syphilis		+	1	3	2.61	0.11
		-	297	167		
HIV		+	0	0	–	–
		-	298	170		
WBC			6.96 ± 2.99	7.67 ± 3.09	2.43	0.02
HB			95.61 ± 24.21	99.91 ± 20.70	1.94	0.05
PLT			198.86 ± 71.99	218.64 ± 72.74	2.85	0.005
NEU			4.84 ± 2.74	5.18 ± 2.81	1.27	0.21
lymph			1.43 ± 1.16	2.12 ± 2.81	3.71	<0.001
ipth			507.75 ± 430.19	361.67 ± 274.27	3.99	<0.001
BUN			25.07 ± 9.90	22.69 ± 11.16	2.39	0.02
CRE			943.21 ± 340.93	709.31 ± 422.84	6.53	<0.001
UA			499.01 ± 134.85	503.24 ± 129.07	0.33	0.74
CA			2.19 ± 0.43	2.19 ± 0.22	0.04	0.97
P			1.97 ± 0.60	1.76 ± 0.62	3.51	<0.001
CHOL			4.37 ± 1.08	4.57 ± 1.25	1.78	0.08
TG			1.69 ± 0.91	1.77 ± 1.27	0.74	0.46
HDL			1.05 ± 0.25	1.08 ± 0.38	0.86	0.40
LDL			2.65 ± 0.77	2.65 ± 0.83	1.75	0.08

WBC, white blood cells; HB, hemoglobin; PLT, platelets; NEU, neutrophile granulocyte; ipth, intact parathyroid hormone; BUN, blood urea nitrogen; CRE, creatinine; UA, uric acid; CA, calcium; P, phosphorus; CHOL, total cholesterol; TG, triglyceride; HDL, high density lipoprotein; LDL, low density lipoprotein.

**Figure 2:**
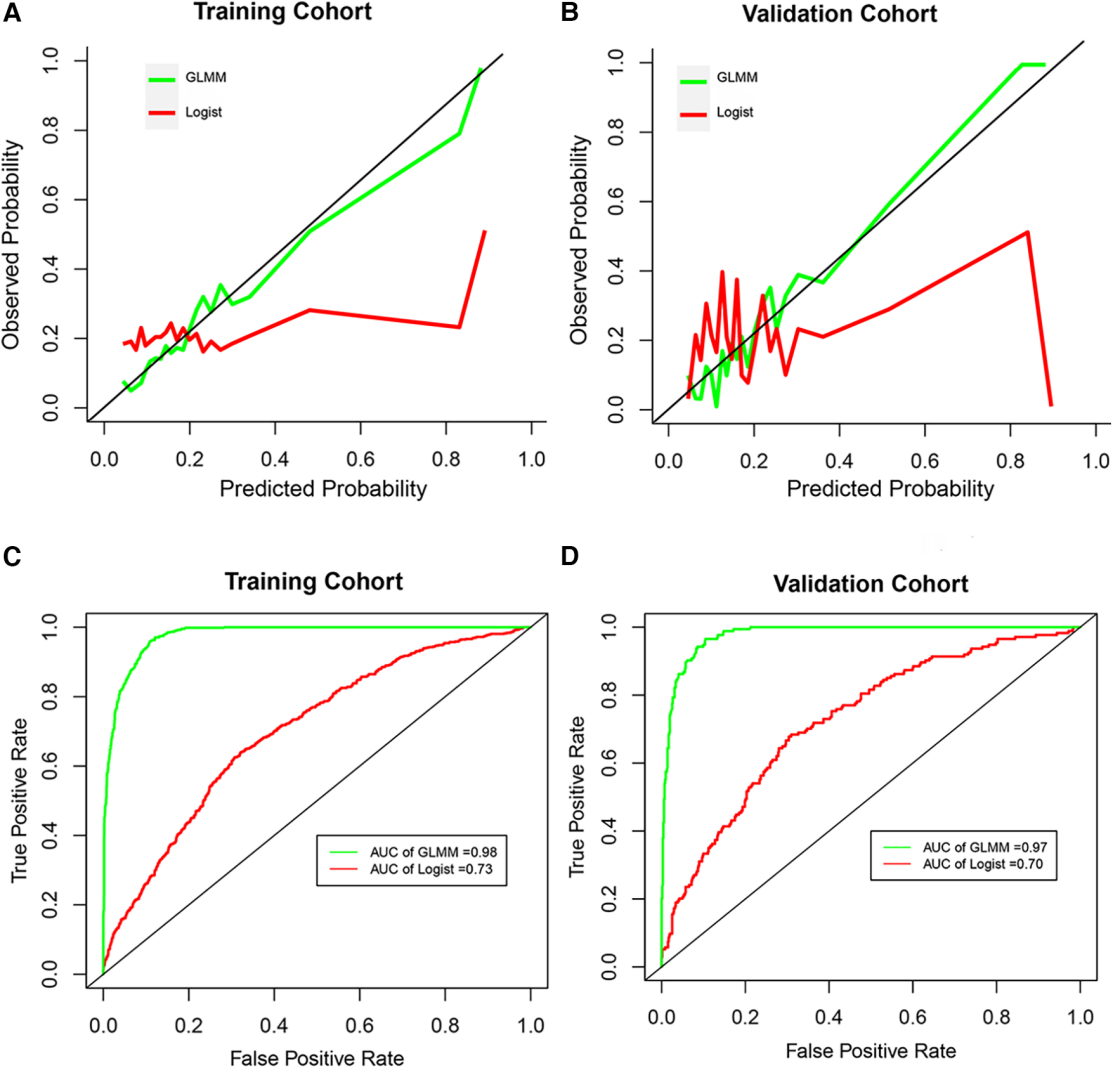
The calibration curve and receiver operation characteristic curve. (**A** and **B**) Represented the calibration curve in the training cohort and validation cohort, respectively. (**C** and **D**) Represented the receiver operation characteristic curve in the training cohort and validation cohort, respectively. GLMM, generalized linear mixed model; Logist, logistic regression model; AUC, area under the receiver operating characteristic curves.

The total classification accuracy for the LRM and GLMM models was 81.4% and 93.9%, respectively. The coefficient of age, diabetes, combined disease, WBC, PLT, NEUT, lymph, P, HBsAg, and HIV was significant (*p *< 0.05) in the LRM. In the GLMM model, the fixed effects of age, diabetes, ipth, CRE, and UA were statistically significant (*p *< 0.05); the random effect of ipth and *P* was significant (*p *< 0.05). The detail of the two models was shown in Table 2. The calibration plot for the GLMM was closer to the ideal curve than the calibration plot for the LRM (Figure 1A). The AUCs of the LRM and GLMM were 0.833 and 0.93, respectively (Figure 2B).

**Table 2:** The comparison of the two models.

	Significant (*p* < 0.05)variables selected by the model (b represented coefficient)		Training set (*N* = 389)				Testing set (*N* = 97)			
			Sensitivity %	Specificity %	Accuracy %	AUC (95% CI)	Sensitivity %	Specificity %	Accuracy %	AUC (95% CI)
Logist	Age (b = 0.029, *p *< 0.001), sex (b = 0.193, *p *= 0.038), *P* (b = 0.413, *p *< 0.001), diabetes (b = 0.46, *p *< 0.001), combined disease (b = 0.232, *p *= 0.007), WBC (b = 0.137, *p *< 0.001), PLT (b = 0.003, *p *< 0.001), NEUT(-0.108, *p *< 0.001), lymph (b = 0.052, *p *= 0.012), HBsAg (b = −0.469, *p *= 0.004), HIV (b = 4.282, *p *< 0.001)		82.3	59.0	81.4	0.73 (0.69–0.77)	81.2	59.1	80.4	0.70 (0.68–0.72)
GLMM	Fixed effects	Age (b = 0.059, *p *< 0.001), diabetes (b = 0.879, *p *= 0.005), ipth (b = 0.001, *p *= 0.002), CRE (b = 0.002, *p *< 0.001), UA (b = −0.002, *p *< 0.001)	95.4	86.7	93.9	0.98 (0.97–0.98)	94.0	85.5	93.1	0.97 (0.97–0.98)
	Random effects	Variation (ipth) = 5.27e-6, *p *< 0.001 Variation (*P*) = 0.47, *p *= 0.005								

AUC, area under the receiver operating characteristic curves; P, the concentration of P; WBC, white blood cells count; NEUT, neutrophil count; CRE, creatinine; UA, uric acid.

**Figure 3:**
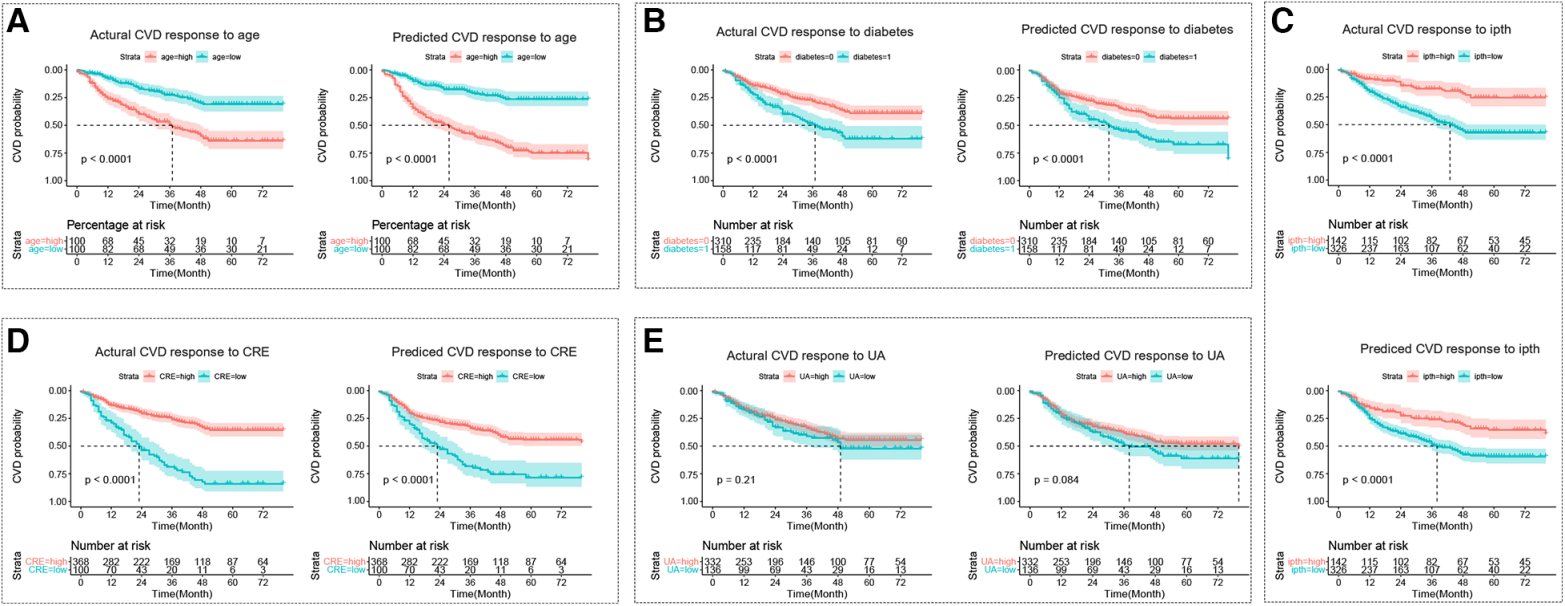
The kaplan-meier curve for the cardiovascular disease response. (**A**–**E**) Represent Kaplan-Meier curves for each sub-classification of age, diabetes, ipth, CRE, and UA, respectively. The cut points for age, ipth, CRE, and UA were 56 years old, 489 pg/ml, 7.53 umol/L, and 1.42 umol/L. In sub-figure b, diabetes = 0 represented no diabetes group, and diabetes = 1 represented diabetes group. CVD, cardiovascular disease; HD, hemodialysis patients; ipth, intact parathyroid hormone; CRE, creatinine; UA, uric acid (UA).

The cut points for age, ipth, CRE, and UA were 56 years old, 489 pg/ml, 7.53 umol/L, and 1.42 umol/L. The cut points divided these continuum factors into two hierarchical levels. The Kaplan-Meier curves for CVD outcome between two hierarchical levels satisfied statistically significant differences (*p *< 0.05) in the actual CVD response and predicted response by the GLMM model, except risk factor of UA (Figure 3).

### 3.1 Causal-relationships among risk factors, ESRD, and CVD

**Figure 4:**
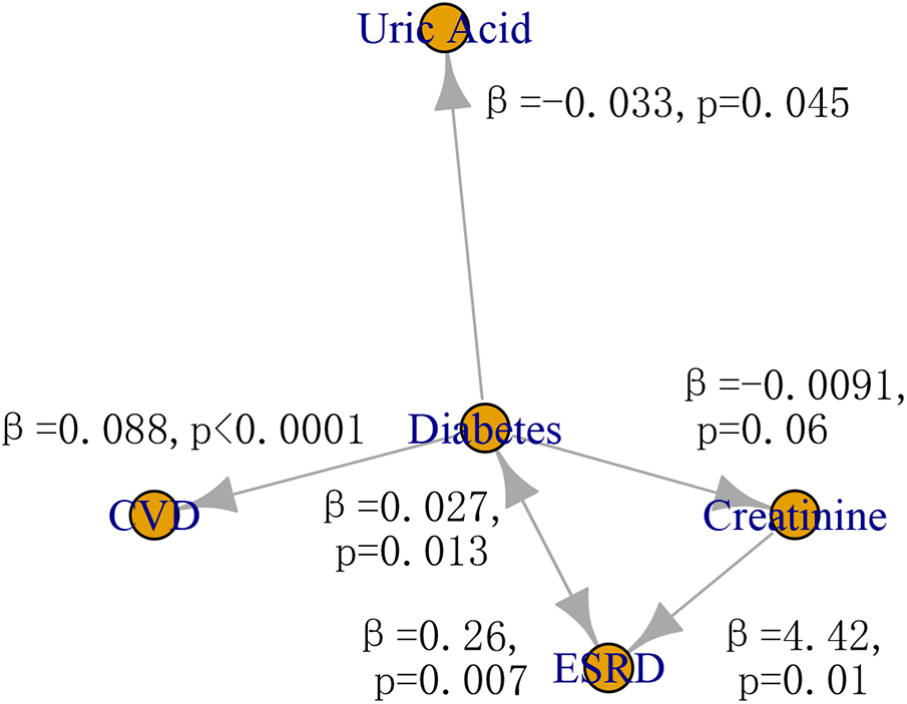
The causal relationship among risk factors, CVD, and ESRD. CVD, cardiovascular disease; ESRD, end-stage renal disease.

The possible causal relationships among risk factors, ESRD, and CVD was shown in Figure 4. The two-sample IVW MR provided evidence for a causal role of diabetes in risk of CVD (*β *=* *0.088, *p *< 0.0001) and ERSD (*β *=* *0.26, *p *= 0.007). In turn, ERSD had a causal role of diabetes (*β *=* *0.027, *p *= 0.013). In addition, diabetes could decrease the level of uric acid (*β *=* *−0.033, *p *= 0.045). The increase of creatinine level had a causal role in risk of ESRD (*β *=* *4.42, *p *= 0.01). There was no evidence of horizontal pleiotropy (*p* of EI > 0.05) in these causal inferences except the causal inference from diabetes to CVD (*p* of EI* *=* *0.045) (Supplementary Tables S2–S6). There was no evidence of heterogeneity except in the causal inference from diabetes to CVD, uric acid, and ESRD (*p* of Q < 0.05). No other causal relationship was found in this study (Supplementary Table S2–S6). The F statistics of SNPs were shown in Supplementary Table S7. The effect size of individual SNP and all SNPs was shown in Supplementary Figure S1.

## 4 Discussion

To the best of our knowledge, this was the first longitudinal cohort study to investigate the causal relationships among risk factors of CVD in patients with ESRD. We developed and validated a clinical GLMM model for selecting risk factors of CVD in patients with hemodialysis based on longitudinal data. Additionally, MR analyses were performed to evaluate the causal relationships among risk factors, CVD, and ESRD. The GLMM model revealed that diabetes, creatinine, UA, and ipth were risk factors for CVD. The MR analyses provided robust genetic evidence supporting the notion that diabetes played a causal role in the relationship between ESRD and CVD, which raised the importance of blood sugar control.

The predictors used in our prediction model integrated various clinical variables and were found to be one of the most important components in estimating the status of the body system. Notably, most of the significant predictors, such as diabetes, ipth, CRE, and UA, were related to metabolism. This finding in consistent with previous studies that have shown metabolic dysfunctions, including diabetes, obesity, and uarthritis, to be involved in aberrations preceding clinically overt CVD (40–43). Furthermore, a recent meta-analysis corroborated these results, revealing association between metabolic dysfunction and CVD (44).

Previous studies have primarily focused on the effects of predictors at a single time point, neglecting the longitudinal effectsin the analysis. In this study, we aimed to examine the potential risk factors for both independence and relevance by using the GLMM model with longitudinal clinical data. Recent studies on degenerative joint disease, COVID-19, and pulmonary parenchymal lesions have revealed that associations that are not apparent in other frameworks can be elucidated using GLMMs (45–47). In our study, compared to the traditional logistic regression model, the GLMM model exhibited higher classification accuracy, indicating a better ability to explain variability. Hence, we selected and identified the results from the GLMM for further analysis in this study. Notably, a significant difference between the two models lies in the predictors. The traditional logistic regression model indicated that predictors such as WBC, PLT, NEUT, and lymph were primarily associated with the immune system. While, in the GLMM, predictors like ipth, CRE, and UA, were predominantly linked to the metabolism system and were not significant in the logistic regression model. Although inflammation has long been considered to be associated with CVD in hemodialysis patients, recent evidence has demonstrated a close relationship between ipth, CRE, UA and CVD (48–51). It is important to note that these predictors had considerable standard deviations, implying substantial interindividual variability, which poses a challenge to the consistency of the results. This variability might explain why the effects of ipth and CRE on CVD in HD patients show inconsistent directions, as both negative (26, 52, 53) and positive (54–56) associations have been reported. On the other hand, clinical data inherently exhibits universal and multidimensional variability due to factors such as exercise, diet, psychology, and some random or non-random noise. However, longitudinal analysis can help “minimized” this variability by capturing diverse patterns of intra- and/or inter-personal variability (57). Therefore, even though no significant differences were observed in UA levels at the baseline, the GLMM revealed UA as a significant predictor (*p *< 0.05). This finding is consistent with a recent meta study that a robust and independent association between elevated UA levels and lower risk of cardiovascular mortality in maintenance hemodialysis patients (58).

In addition to assessing CVD occurrence, the performance of our GLMM was evaluated by the Kaplan-Meier curves. It was observed that the Kaplan-Meier curve of the CVD occurrence or no occurrence predicted by our GLMM was similar to the curve of the CVD status observed by a physician. In addition, the utility of the GLMM was convincingly supported by the significant differences in Kaplan-Meier curves across different levels of age, diabetes, ipth, CRE, and UA, all of which were identified as significant predictors by the GLMM. Our results showed that patients with older age, diabetes, lower ipth, lower CRE, or lower UA were at a higher risk of CVD. The causal role of old age and diabetes have long been recognized, and the possible mechanisms have been extensively studies (59, 60). There is ample evidence to suggest that lower ipth, CRE, or UA is associated with a loss of autonomy or inflammation (61–63). However, the relationships among these risk factors, CVD and ESRD remain unclear.

Our MR analyses showed that diabetes played an important bridging role among them, suggesting the importance of blood glucose control in preventing both CVD and ESRD. Consistent with previous MR results, diabetes has causal effect on cardiovascular injury and kidney disease (64, 65). Recent MR studies from different cohorts have reported no causal effect of uric acid type 2 diabetes, although a significant association have been widely reported, which is in line with our result (66–68). Unlike previous MR studies, bidirectional causal effect was considered to identify some new causal effects in this study. Through bidirectional MR analysis, diabetes was also found to have a causal role in the decrease of uric acid and creatinine, which were risk factors identified by the longitudinal analysis. Our findings may help explain the associations between diabetes and clinical factors such as uric acid and creatinine observed in previous observational studies (69, 70).

Despite the causal relationships between the risk factors and CVD in HD, this study has several limitations. Firstly, this is a retrospective study, and several clinical variables could not be measured. For example, the absence of various proinflammatory cytokines, such as interleukin-1 beta, interleukin 6, and transforming growth factor beta (71), makes it impossible to further explore the relationship between the inflammatory response and the risk factors identified in this research. In addition, the nutritional status is also an important indicator, as dialysis patients often face the problem of inadequate nutrition intake, and nutrition can potentially affect patients' quality of life and treatment outcomes (72). Secondly, although subjects were selected based on the inclusion criteria, the heterogeneity across patients due to factors like medication, lifestyle habits, and uncounted confounding factors, might yeild a null hypothesis. Thirdly, we did not classify the type of CVD, but the impact of risk factors might differ across CVD subtypes. Future studies with sufficiently large sample sizes can be carried out to explore the difference among CVD subtypes. Currently, our model assists clinicians in selecting targeted agents in advance to enable appropriate treatment choices for HD patients. Finally, horizontal pleiotropy and heterogeneity resulting from population variances in GWAS statistics can affect the MR results. In order to provide consistent and robust causal estimation, multiple sensitivity analyses, such as simple median, weighted median, and MR-Egger methods, were employed in this study. In future, larger sample size studies are needed to validate these issues.

In conclusion, our longitudinal model revealed that old age, diabetes, depressed ipth, CRE, and UA were risk factors for CVD in HD patients, with diabetes playing an important bridging role among them. Effective blood glucose control is crucial for preventing CVD in HD patients.

## Data Availability

The original contributions presented in the study are included in the article/Supplementary Material, further inquiries can be directed to the corresponding authors.
