# Supplementary material for: Causal factors of cardiovascular disease in end-stage renal disease with maintenance hemodialysis: a longitudinal and Mendelian randomization study
Source: Front Cardiovasc Med. 2024 Jul 18;11:1306159. doi: 10.3389/fcvm.2024.1306159 (PMC11291196; doi:10.3389/fcvm.2024.1306159)
Supplement: Supplementary file 1 [file Datasheet1.docx]

**Supplementary Table 1.** The basis information of the selected GWAS statistics.

| ID | Trait | Population | Sex | Sample Size | Number of SNP |
| --- | --- | --- | --- | --- | --- |
| ebi-a-GCST008031 | End-stage renal disease | Hispanic or Latin American | NA | 16405 | 11547772 |
| finn-b-FG_CVD | Cardiovascular diseases | European | Males and Females | 218792 | 16380466 |
| finn-b-T2D_WIDE | Type 2 diabetes | European | Males and Females | 202046 | 16380418 |
| met-a-309 | Creatinine | European | Males and Females | 7810 | 2545671 |
| Bjj-a-57 | Uric acid | East Asian | Males and Females | 109029 | 6108953 |

| Analysis | β | SE | *p* | Horizontal Pleiotropy: EI | Heterogeneity: Q |
| --- | --- | --- | --- | --- | --- |
| Diabetes VS ESRD: exposure ID=ebi-a-GCST008031, outcome ID=ubm-a-2699 | | | | | |
| MR Egger | 0.105 | 0.230 | 0.65 | 0.067, *p*=0.59 | 27.91, *p*=0.16 |
| Weighted Median | 0.206 | 0.143 | 0.15 |  |  |
| Inverse Variance Weighted | 0.261 | 0.097 | 0.007 |  | 28.83, *p*=0.19 |
| Simple Mode | 0.225 | 0.295 | 0.45 |  |  |
| Weighted Mode | 0.088 | 0.173 | 0.61 |  |  |
| creatinine VS ESRD: exposure ID=met-a-309, outcome ID=ubm-a-2699 | | | | | |
| MR Egger | 2.02 | 4.01 | 0.62 | 0.022, *p*=0.51 | 6.27, *p*=0.85 |
| Weighted Median | 4.06 | 2.56 | 0.11 |  |  |
| Inverse Variance Weighted | 4.42 | 1.79 | 0.01 |  | 6.72, *p*=0.87 |
| Simple Mode | 8.76 | 4.26 | 0.06 |  |  |
| Weighted Mode | 6.89 | 3.21 | 0.05 |  |  |
| UA VS ESRD: exposure ID=bbj-a-57, outcome ID=ubm-a-2699 | | | | | |
| MR Egger | -0.18 | 0.23 | 0.45 | 0.0070, *p*=0.62 | 30.27, *p*=0.87 |
| Weighted Median | -0.18 | 0.21 | 0.39 |  |  |
| Inverse Variance Weighted | -0.08 | 0.13 | 0.54 |  | 30.52, *p*=0.88 |
| Simple Mode | 0.17 | 0.41 | 0.68 |  |  |
| Weighted Mode | 0.23 | 0.27 | 0.39 |  |  |
| CVD VS ESRD: exposure ID=finn-b-FG_CVD, outcome ID=ubm-a-2699 | | | | | |
| MR Egger | 0.06 | 1.47 | 0.97 | 0.019, *p*=0.84 | 11.10, *p*=0.43 |
| Weighted Median | 0.21 | 0.34 | 0.54 |  |  |
| Inverse Variance Weighted | 0.35 | 0.25 | 0.16 |  | 11.14, *p*=0.51 |
| Simple Mode | 0.13 | 0.58 | 0.82 |  |  |
| Weighted Mode | 0.21 | 0.57 | 0.72 |  |  |

**Supplementary Table 2.** Causal effect of diabetes, creatinine, uric acid, and CVD on ESRD.

Abbreviations: CVD, cardiovascular disease; ESRD, end stage renal disease; SE, stand error.

**Supplementary Table 3.** Causal effect of diabetes, creatinine, uric acid, and ESRD on CVD.

| Analysis | β | SE | *p* | Horizontal Pleiotropy: EI | Heterogeneity: Q |
| --- | --- | --- | --- | --- | --- |
| Diabetes VS CVD: exposure ID=finn-b-T2D_WIDE, outcome ID=finn-b-FG_CVD | | | | | |
| MR Egger | -0.0007 | 0.044 | 0.98 | 0.011, *p*=0.045 | 60.64, *p*=5.13e-4 |
| Weighted Median | 0.064 | 0.023 | 0.0053 |  |  |
| Inverse Variance Weighted | 0.082 | 0.021 | 9.81e-5 |  | 69.77, *p*=5.21e-5 |
| Simple Mode | 0.12 | 0.045 | 0.012 |  |  |
| Weighted Mode | 0.049 | 0.027 | 0.085 |  |  |
| creatinine VS CVD: exposure ID=met-a-309, outcome ID=finn-b-FG_CVD | | | | | |
| MR Egger | -1.67 | 0.76 | 0.048 | 0.013, *p*=0.039 | 10.99, *p*=0.53 |
| Weighted Median | 0.093 | 0.48 | 0.85 |  |  |
| Inverse Variance Weighted | -0.086 | 0.37 | 0.81 |  | 16.35, *p*=0.23 |
| Simple Mode | 0.34 | 0.80 | 0.68 |  |  |
| Weighted Mode | 0.16 | 0.79 | 0.84 |  |  |
| UA VS CVD: exposure ID=bbj-a-57, outcome ID=finn-b-FG_CVD | | | | | |
| MR Egger | -0.069 | 0.059 | 0.25 | 0.0080, *p*=0.0038 | 100.84, *p*=3.67e-7 |
| Weighted Median | -0.024 | 0.034 | 0.47 |  |  |
| Inverse Variance Weighted | 0.027 | 0.038 | 0.48 |  | 111.81, *p*=1.75e-8 |
| Simple Mode | 0.032 | 0.074 | 0.66 |  |  |
| Weighted Mode | -0.0089 | 0.030 | 0.77 |  |  |
| ESRD VS CVD: exposure ID=ebi-a-GCST008031, outcome ID=finn-b-FG_CVD | | | | | |
| MR Egger | 0.022 | 0.016 | 0.19 | -0.0084, *p*=0.19 | 23.33, *p*=0.27 |
| Weighted Median | 0.0045 | 0.010 | 0.66 |  |  |
| Inverse Variance Weighted | 0.0024 | 0.0074 | 0.74 |  | 25.52, *p*=0.23 |
| Simple Mode | -0.012 | 0.017 | 0.48 |  |  |
| Weighted Mode | 0.0045 | 0.011 | 0.69 |  |  |

**Supplementary Table 4.** Causal effect of ESRD, CVD, creatinine, and uric acid on diabetes.

| Analysis | β | SE | *p* | Horizontal Pleiotropy: EI | Heterogeneity: Q |
| --- | --- | --- | --- | --- | --- |
| ESRD VS Diabetes: exposure ID=ebi-a-GCST008031, outcome ID=finn-b-T2D_WIDE | | | | | |
| MR Egger | 0.0098 | 0.026 | 0.71 | 0.026, *p*=0.10 | 22.33, *p*=0.34 |
| Weighted Median | 0.012 | 0.017 | 0.49 |  |  |
| Inverse Variance Weighted | 0.027 | 0.012 | 0.021 |  | 26.90, *p*=0.17 |
| Simple Mode | 0.054 | 0.026 | 0.052 |  |  |
| Weighted Mode | 0.011 | 0.017 | 0.54 |  |  |
| CVD VS Diabetes : exposure ID=finn-b-FG_CVD, outcome ID=finn-b-T2D_WIDE | | | | | |
| MR Egger | -0.85 | 0.67 | 0.23 | 0.084, *p*=0.084 | 57.79, *p*=1.30e-7 |
| Weighted Median | 0.21 | 0.093 | 0.021 |  |  |
| Inverse Variance Weighted | 0.37 | 0.15 | 0.013 |  | 73.33, *p*=4.79e-10 |
| Simple Mode | 0.16 | 0.13 | 0.24 |  |  |
| Weighted Mode | 0.17 | 0.12 | 0.17 |  |  |
| creatinine VS Diabetes: exposure ID=met-a-309, outcome ID=finn-b-T2D_WIDE | | | | | |
| MR Egger | -0.37 | 1.34 | 0.79 | 0.0018, *p*=0.65 | 5.26, *p*=0.73 |
| Weighted Median | 0.19 | 0.75 | 0.80 |  |  |
| Inverse Variance Weighted | 0.19 | 0.56 | 0.73 |  | 5.49, *p*=0.79 |
| Simple Mode | 0.80 | 1.18 | 0.51 |  |  |
| Weighted Mode | 0.038 | 0.87 | 0.96 |  |  |
| UA VS Diabetes: exposure ID=bbj-a-57, outcome ID=finn-b-T2D_WIDE | | | | | |
| MR Egger | 0.027 | 0.088 | 0.75 | -6.78e-5, *p*=0.99 | 80.42, *p*=0.00016 |
| Weighted Median | 0.059 | 0.060 | 0.32 |  |  |
| Inverse Variance Weighted | 0.027 | 0.054 | 0.62 |  | 80.42 *p*=0.00023 |
| Simple Mode | 0.013 | 0.11 | 0.91 |  |  |
| Weighted Mode | 0.013 | 0.052 | 0.81 |  |  |

| Analysis | β | SE | *p* | Horizontal Pleiotropy: EI | Heterogeneity: Q |
| --- | --- | --- | --- | --- | --- |
| ERSD VS Creatinine: exposure ID=ebi-a-GCST008031, outcome ID=met-a-309 | | | | | |
| MR Egger | -0.013 | 0.01 | 0.23 | 0.0030, *p*=0.27 | 13.38, *p*=0.42 |
| Weighted Median | -0.0019 | 0.0033 | 0.57 |  |  |
| Inverse Variance Weighted | -0.00031 | 0.0023 | 0.89 |  | 14.72, *p*=0.40 |
| Simple Mode | -0.005 | 0.005 | 0.34 |  |  |
| Weighted Mode | -0.0031 | 0.0047 | 0.52 |  |  |
| CVD VS Creatinine : exposure ID=finn-b-FG_CVD, outcome ID=met-a-309 | | | | | |
| MR Egger | -0.062 | 0.061 | 0.33 | 0.0018, *p*=0.65 | 5.26, *p*=0.73 |
| Weighted Median | -0.0072 | 0.013 | 0.56 |  |  |
| Inverse Variance Weighted | -0.014 | 0.0093 | 0.14 |  | 5.49, *p*=0.79 |
| Simple Mode | -0.01 | 0.018 | 0.95 |  |  |
| Weighted Mode | -0.0025 | 0.017 | 0.88 |  |  |
| Diabetes VS Creatinine: exposure ID=finn-b-T2D_WIDE, outcome ID=met-a-309 | | | | | |
| MR Egger | -0.00089 | 0.012 | 0.941 | 0.0011, *p*=0.46 | 23.53, *p*=0.052 |
| Weighted Median | -0.0051 | 0.0054 | 0.35 |  |  |
| Inverse Variance Weighted | -0.0091 | 0.0048 | 0.060 |  | 24.50, *p*=0.057 |
| Simple Mode | -0.0011 | 0.0094 | 0.91 |  |  |
| Weighted Mode | -0.0031 | 0.0057 | 0.599 |  |  |
| UA VS Creatinine: exposure ID=bbj-a-57, outcome ID=met-a-309 | | | | | |
| MR Egger | 0.0049 | 0.0082 | 0.55 | -0.015, *p*=0.16 | 59.27, *p*=6.48e-10 |
| Weighted Median | 0.0041 | 0.0071 | 0.56 |  |  |
| Inverse Variance Weighted | 0.00049 | 0.0049 | 0.92 |  | 76.83, *p*=6.86e-13 |
| Simple Mode | -0.0072 | 0.013 | 0.59 |  |  |
| Weighted Mode | 0.0022 | 0.0063 | 0.72 |  |  |

**Supplementary Table 5.** Causal effect of ESRD, CVD, diabetes, and uric acid on creatinine.

**Supplementary Table 6.** Causal effect of ESRD, CVD, diabetes, and creatinine on uric acid.

| Analysis | β | SE | *p* | Horizontal Pleiotropy: EI | Heterogeneity: Q |
| --- | --- | --- | --- | --- | --- |
| ESRD VS UA: exposure ID=ebi-a-GCST008031, outcome ID=bbj-a-57 | | | | | |
| MR Egger | -0.031 | 0.016 | 0.089 | -0.0095, *p*=0.085 | 14.36, *p*=0.16 |
| Weighted Median | -0.0064 | 0.0085 | 0.46 |  |  |
| Inverse Variance Weighted | -0.0019 | 0.0071 | 0.79 |  | 19.61, *p*=0.06 |
| Simple Mode | -0.013 | 0.015 | 0.42 |  |  |
| Weighted Mode | -0.0098 | 0.011 | 0.39 |  |  |
| CVD VS UA : exposure ID=finn-b-FG_CVD, outcome ID=bbj-a-57 | | | | | |
| MR Egger | 0.34 | 0.56 | 0.55 | -0.023, *p*=0.55 | 61.38, *p*=2.49e-10 |
| Weighted Median | 0.1 | 0.043 | 0.81 |  |  |
| Inverse Variance Weighted | 0.0048 | 0.067 | 0.94 |  | 64.28 *p*=1.98e-10 |
| Simple Mode | -0.023 | 0.077 | 0.77 |  |  |
| Weighted Mode | -0.016 | 0.074 | 0.83 |  |  |
| Diabetes VS UA: exposure ID=finn-b-T2D_WIDE, outcome ID=bbj-a-57 | | | | | |
| MR Egger | -0.14 | 0.050 | 0.0088 | -0.013, *p*=0.29 | 43.27, *p*=1.88e-3 |
| Weighted Median | -0.055 | 0.018 | 0.0022 |  |  |
| Inverse Variance Weighted | -0.033 | 0.017 | 0.049 |  | 55.24, *p*=6.52e-5 |
| Simple Mode | -0.048 | 0.042 | 0.26 |  |  |
| Weighted Mode | -0.071 | 0.023 | 0.0058 |  |  |
| Creatinine VS UA: exposure ID=met-a-309, outcome ID=bbj-a-57 | | | | | |
| MR Egger | 1.69 | 1.43 | 0.27 | -0.015, *p*=0.16 | 59.27, *p*=6.47e-10 |
| Weighted Median | 0.092 | 0.32 | 0.77 |  |  |
| Inverse Variance Weighted | -0.29 | 0.66 | 0.66 |  | 76.83, *p*=6.86e-13 |
| Simple Mode | -0.69 | 0.59 | 0.27 |  |  |
| Weighted Mode | 0.17 | 0.320 | 0.60 |  |  |

**Supplementary Table 7.** The information of selected instruments in MR analysis

| SNP in causal effect of type 2 diabetes on ESRD | | | | |
| --- | --- | --- | --- | --- |
| Name | Chrome | β | SE | F statistic |
| rs1046317 | 4 | 0.0853 | 0.0134 | 259.18 |
| rs10830963 | 11 | 0.0871 | 0.0137 | 260.02 |
| rs10882099 | 10 | -0.0718 | 0.0131 | 192.39 |
| rs10965246 | 9 | -0.1211 | 0.0183 | 283.72 |
| rs11257658 | 10 | 0.0914 | 0.0149 | 242.91 |
| rs11712037 | 3 | -0.0951 | 0.0174 | 192.22 |
| rs1815311 | 6 | 0.0769 | 0.0134 | 213.17 |
| rs2237897 | 11 | -0.1652 | 0.0242 | 307.54 |
| rs2394972 | 6 | 0.0932 | 0.0149 | 250.75 |
| rs28624681 | 9 | 0.0909 | 0.0144 | 256.36 |
| rs2943650 | 2 | 0.0882 | 0.0136 | 268.84 |
| rs3104368 | 6 | 0.2363 | 0.0185 | 1092.51 |
| rs34298980 | 6 | -0.0817 | 0.0141 | 225.61 |
| rs34872471 | 10 | 0.2747 | 0.0166 | 1842.43 |
| rs3887925 | 3 | 0.0792 | 0.0131 | 233.42 |
| rs55993634 | 16 | -0.1441 | 0.0233 | 249.50 |
| rs56348580 | 12 | -0.0824 | 0.0146 | 206.39 |
| rs7018475 | 9 | 0.1085 | 0.0146 | 354.37 |
| rs7109575 | 11 | -0.0921 | 0.0154 | 230.36 |
| rs73193383 | 4 | 0.1335 | 0.0243 | 195.46 |
| rs7633675 | 3 | 0.0943 | 0.0141 | 283.19 |
| rs77655131 | 7 | 0.0954 | 0.017 | 203.40 |
| rs9273401 | 6 | 0.2845 | 0.0203 | 1329.62 |
| rs9348441 | 6 | 0.13 | 0.0139 | 559.69 |
| rs9937053 | 16 | 0.1275 | 0.0132 | 598.80 |
| SNP in causal effect of type 2 diabetes on creatinine | | | | |
| rs1046317 | 4 | 0.0853 | 0.0134 | 259.19 |
| rs10830963 | 11 | 0.0871 | 0.0137 | 260.01 |
| rs10882099 | 10 | -0.0718 | 0.0131 | 192.39 |
| rs10965246 | 9 | -0.1211 | 0.0183 | 283.71 |
| rs2237897 | 11 | -0.1652 | 0.0242 | 307.53 |
| rs2943650 | 2 | 0.0882 | 0.0136 | 268.84 |
| rs34872471 | 10 | 0.2747 | 0.0166 | 1842.44 |
| rs3887925 | 3 | 0.0792 | 0.0131 | 233.42 |
| rs7018475 | 9 | 0.1085 | 0.0146 | 354.38 |
| rs7109575 | 11 | -0.0921 | 0.0154 | 230.36 |
| rs73193383 | 4 | 0.1335 | 0.0243 | 195.46 |
| rs7633675 | 3 | 0.0943 | 0.0141 | 283.19 |
| rs9348441 | 6 | 0.13 | 0.0139 | 559.69 |
| rs9937053 | 16 | 0.1275 | 0.0132 | 598.81 |
| SNP in causal effect of type 2 diabetes on CVD | | | | |
| rs1046317 | 4 | 0.0853 | 0.0134 | 259.19 |
| rs10830963 | 11 | 0.0871 | 0.0137 | 260.016 |
| rs10882099 | 10 | -0.0718 | 0.0131 | 192.39 |
| rs10965246 | 9 | -0.1211 | 0.0183 | 283.72 |
| rs112108223 | 12 | -0.275 | 0.0453 | 252.03 |
| rs11257658 | 10 | 0.0914 | 0.0149 | 242.92 |
| rs11712037 | 3 | -0.0951 | 0.0174 | 192.22 |
| rs1815311 | 6 | 0.0769 | 0.0134 | 213.17 |
| rs2237897 | 11 | -0.1652 | 0.0242 | 307.54 |
| rs2254021 | 17 | 0.0739 | 0.0134 | 195.25 |
| rs2394972 | 6 | 0.0932 | 0.0149 | 250.75 |
| rs28624681 | 9 | 0.0909 | 0.0144 | 256.36 |
| rs2943650 | 2 | 0.0882 | 0.0136 | 268.84 |
| rs3104368 | 6 | 0.2363 | 0.0185 | 1092.51 |
| rs3104368 | 6 | 0.2363 | 0.0185 | 1092.51 |
| rs34298980 | 6 | -0.0817 | 0.0141 | 225.61 |
| rs34872471 | 10 | 0.2747 | 0.0166 | 1842.43 |
| rs3887925 | 3 | 0.0792 | 0.0131 | 233.42 |
| rs45551238 | 20 | -0.2201 | 0.0306 | 347.08 |
| rs55993634 | 16 | -0.1441 | 0.0233 | 249.50 |
| rs56348580 | 12 | -0.0824 | 0.0146 | 206.39 |
| rs590015 | 6 | 0.0726 | 0.0131 | 197.12 |
| rs7018475 | 9 | 0.1085 | 0.0146 | 354.38 |
| rs7109575 | 11 | -0.0921 | 0.0154 | 230.36 |
| rs73193383 | 4 | 0.1335 | 0.0243 | 195.46 |
| rs7633675 | 3 | 0.0943 | 0.0141 | 283.19 |
| rs76895963 | 12 | -0.3873 | 0.0404 | 696.13 |
| rs77655131 | 7 | 0.0954 | 0.017 | 203.40 |
| rs78470967 | 12 | -0.198 | 0.0341 | 224.75 |
| rs9273401 | 6 | 0.2845 | 0.0203 | 1329.62 |
| rs9348441 | 6 | 0.13 | 0.0139 | 559.69 |
| rs9937053 | 16 | 0.1275 | 0.0132 | 598.80 |
| SNP in causal effect of type 2 diabetes on UA | | | | |
| rs1046317 | 4 | 0.0853 | 0.0134 | 259.18 |
| rs10830963 | 11 | 0.0871 | 0.0137 | 260.01 |
| rs10882099 | 10 | -0.0718 | 0.0131 | 192.39 |
| rs10965246 | 9 | -0.1211 | 0.0183 | 283.71 |
| rs11257658 | 10 | 0.0914 | 0.0149 | 242.91 |
| rs11712037 | 3 | -0.0951 | 0.0174 | 192.22 |
| rs1815311 | 6 | 0.0769 | 0.0134 | 213.17 |
| rs2237897 | 11 | -0.1652 | 0.0242 | 307.54 |
| rs2254021 | 17 | 0.0739 | 0.0134 | 195.25 |
| rs2394972 | 6 | 0.0932 | 0.0149 | 250.75 |
| rs28624681 | 9 | 0.0909 | 0.0144 | 256.36 |
| rs2943650 | 2 | 0.0882 | 0.0136 | 268.84 |
| rs34298980 | 6 | -0.0817 | 0.0141 | 225.61 |
| rs34872471 | 10 | 0.2747 | 0.0166 | 1842.43 |
| rs3887925 | 3 | 0.0792 | 0.0131 | 233.419 |
| rs55993634 | 16 | -0.1441 | 0.0233 | 249.50 |
| rs590015 | 6 | 0.0726 | 0.0131 | 197.11 |
| rs7018475 | 9 | 0.1085 | 0.0146 | 354.377 |
| rs7109575 | 11 | -0.0921 | 0.0154 | 230.364 |
| rs73193383 | 4 | 0.1335 | 0.0243 | 195.46 |
| rs7633675 | 3 | 0.0943 | 0.0141 | 283.19 |
| rs9348441 | 6 | 0.13 | 0.0139 | 559.69 |
| rs9937053 | 16 | 0.1275 | 0.0132 | 598.80 |
| SNP in causal effect of creatinine on ESRD | | | | |
| rs10426415 | 19 | 0.0086 | 0.0018 | 22.83 |
| rs10519445 | 15 | -0.0045 | 9.00E-04 | 25.00 |
| rs11054328 | 12 | 0.0088 | 0.0018 | 23.90 |
| rs1116866 | 1 | -0.0094 | 0.0018 | 27.27 |
| rs12175103 | 6 | 0.0048 | 9.00E-04 | 28.44 |
| rs1498350 | 1 | -0.0045 | 0.001 | 20.25 |
| rs2042499 | 7 | 0.0052 | 9.00E-04 | 33.38 |
| rs2289804 | 10 | 0.0143 | 0.0027 | 28.05 |
| rs394282 | 1 | -0.0163 | 0.0033 | 24.39 |
| rs4460629 | 1 | 0.0045 | 0.001 | 20.25 |
| rs6663614 | 1 | -0.0104 | 0.0022 | 22.34 |
| rs9312777 | 5 | -0.0043 | 9.00E-04 | 22.82 |
| rs9456538 | 6 | -0.0082 | 0.0018 | 20.75 |
| SNP in causal effect of ESRD on diabetes | | | | |
| rs111335814 | 14 | 0.762936 | 0.1666 | 20.97 |
| rs114389188 | 3 | 0.399001 | 0.0848364 | 22.12 |
| rs114425659 | 4 | 0.622839 | 0.126672 | 24.17 |
| rs114874628 | 15 | 0.737986 | 0.154498 | 22.81 |
| rs116247754 | 4 | 0.922804 | 0.189903 | 23.61 |
| rs12135814 | 1 | 0.404232 | 0.0872032 | 21.48 |
| rs12963285 | 18 | -0.298114 | 0.0608801 | 23.97 |
| rs136160 | 22 | -0.231904 | 0.0488567 | 22.53 |
| rs16951881 | 17 | 0.290575 | 0.0620835 | 21.90 |
| rs2512417 | 8 | 0.556835 | 0.120023 | 21.52 |
| rs27547 | 5 | -0.23479 | 0.0513745 | 20.88 |
| rs62023754 | 16 | 0.376524 | 0.0813056 | 21.45 |
| rs62507011 | 7 | 0.47075 | 0.101217 | 21.63 |
| rs6906363 | 6 | -0.235427 | 0.0493172 | 22.78 |
| rs7241420 | 18 | -0.349844 | 0.0723338 | 23.39 |
| rs73044536 | 2 | 0.220557 | 0.0475104 | 21.55 |
| rs738329 | 22 | 0.294403 | 0.0606222 | 23.58 |
| rs7869904 | 9 | -0.371229 | 0.0810696 | 20.97 |
| rs8050506 | 16 | 0.249872 | 0.051069 | 23.94 |
| rs924828 | 17 | -0.280233 | 0.0578935 | 23.43 |
| rs9469220 | 6 | -0.212286 | 0.0449684 | 22.28 |
| rs9847000 | 3 | -0.226556 | 0.0489113 | 21.45 |

F statistics of single nucleotide polymorphisms were calculated to assess whether instrumental variables were sufficiently strong to avoid bias in estimating causal effects referred to Yeung et al (Diabetes Care, 2018, 41(9):1911-1997). Abbreviations: MR, Mendelian randomization; CVD, cardiovascular disease; ESRD, end stage renal disease; SE, stand error; SNP, single nucleotide polymorphism; UA, uric acid.

**
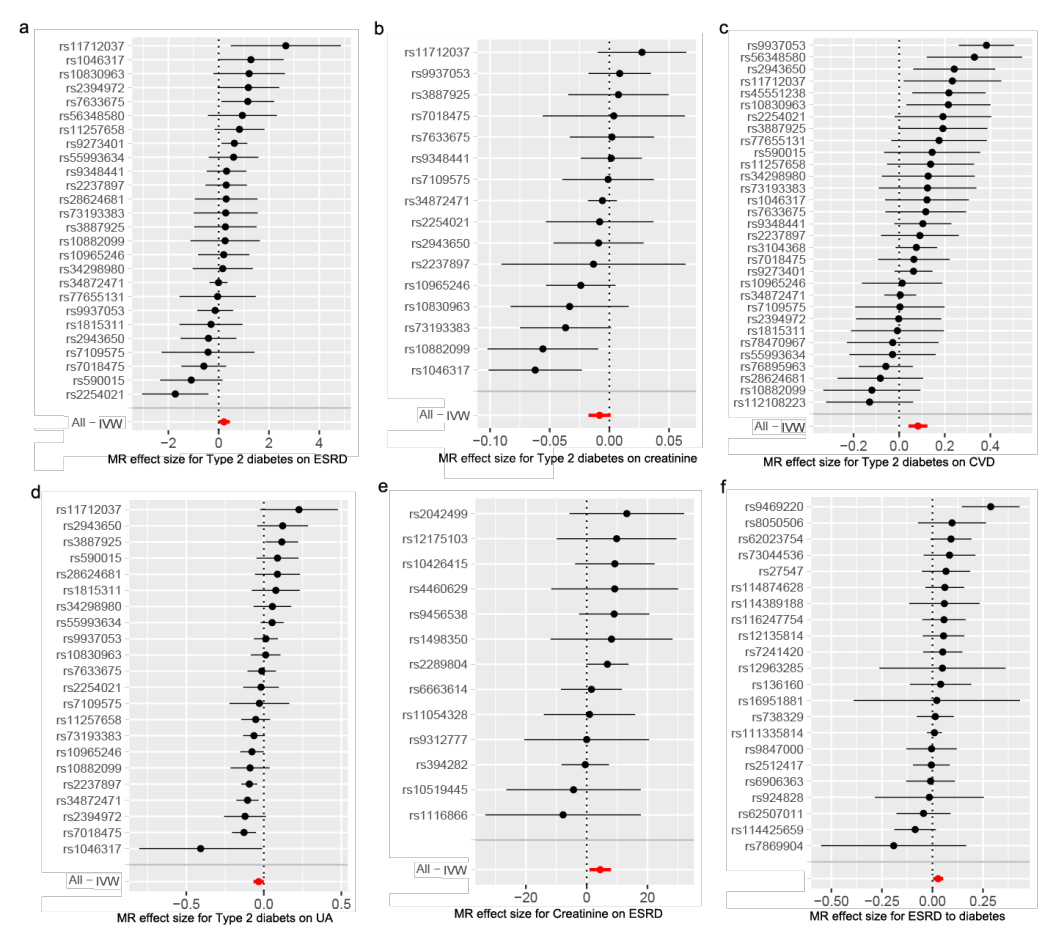
Supplementary Figure 1.** The effect size of individual SNP and whole SNPs. a, b, c, d represented effect size for type 2 diabetes on ESRD, creatinine, CVD, and UA, respectively. E represented effect size for creatinine on ESRD. F represented effect size for ESRD to diabetes. Abbreviations: SNP, single-nucleotide polymorphisms; ESRD, end-stage renal disease; CVD, cardiovascular disease; UA, uric acid.
